# Preliminary Mechanical Evaluation of Grouting Concrete as a Protective Layer for Tunnelling

**DOI:** 10.3390/ma16144957

**Published:** 2023-07-12

**Authors:** Yi Lu, Tong Wan, Xiangyun Huang, Jiahui Lu, Shan Lin, Xingzhong Nong

**Affiliations:** 1 School of Civil Engineering, Guangzhou University, Guangzhou 510006, China; luyi@gzhu.edu.cn (Y.L.); 2112116009@e.gzhu.edu.cn (T.W.); 2 Earthquake Engineering Research and Test Center, Guangzhou 510405, China; 3 Department of Civil Engineering, Xi’an University of Architecture and Technology (XAUAT), Xi’an 710055, China; kelvin@xauat.edu.cn; 4 Guangzhou Metro Design & Research Institute Co., Ltd., Guangzhou 510010, China; linshan@dtsjy.com (S.L.); nongxingzhong@gmdi.cn (X.N.)

**Keywords:** tunnelling, dynamic load, concrete, porous material, unconfined compressive strength

## Abstract

The aim of this study is to introduce a protective layer to safeguard tunnel structures. In practice, one viable approach to create this protective layer between the tunnel structure and surrounding rocks is to pump the material during tunnel construction. The primary components of the proposed material are porous sand, rubber, and cement. Static and dynamic experiments were conducted to assess the unconfined compressive strength (UCS), flexural stiffness, and compaction resistance at various mixing ratios. The results indicate that the addition of porous sand decreases the UCS compared to the solid sand under similar mixing conditions. The addition of rubber offers the elasticity, thereby enhancing the compaction resistance. However, increasing the rubber content compromises UCS. Furthermore, this study presents a linear equation to predict the 7-day UCS, which can be used as a rapid estimation for UCS, flexural stiffness, and compaction resistance of the proposed material. It is important to note that this study only investigates the fundamental mechanical properties of the proposed material, and further comprehensive research is necessary to fully understand its workability, durability, and other behaviour before practical application.

## 1. Introduction

The development of tunnelling projects is rapid in some cities due to the high demand from public transportation [1,2,3]. However, it is usually difficult for countries such as Japan and China to develop underground tunnel projects, as they will undergo adverse geological conditions, such as earthquakes, liquefaction, and creep [4,5,6,7]. As a result, the damage to tunnels due to the seismic action will include tunnel lining deformation, tunnel entrance crack, and other types of damages [8,9,10,11,12,13,14]. The actual mechanism of the soil and rock are far more complicated than the understanding [15]. In fact, when the tunnel is damaged in the geological disaster, it is difficult and almost impossible to be repaired [16]. Table 1 lists some damaged tunnel incidents after earthquake events, and it has been widely agreed that it is necessary to consider the seismic design for tunnel construction to minimise the earthquake effect [17,18,19,20]. 

The seismic considerations for tunnel construction can be generally divided into three categories [21,22,23,24]. In general, the first category is to set a shock protective layer between the tunnel lining and the surrounding soils [25,26,27,28]. The second category is to optimize the damping performance of the tunnel. The third category is to reinforce the surrounding soils. Among these categories, the tunnel protective layer is a material between the tunnel structure and the soil. The lining material of the tunnel can be also considered as the damping layer, as it can effectively absorb the energy transmitted by the seismic waves and reduce the dynamic response of the tunnel structure [28,29,30,31]. Zhou et al. [28] found that the damping performance with double local shock absorption layer is the best, and the damping performance of the shock-absorption layer under large earthquakes is better than that under small earthquakes. Ma et al. [31] analysed the tunnel damping layer using the wave theory, showing that the smaller elastic modulus of the damping layer will lead to a better damping effect. The shear modulus of the damping layer also has an effect on the damping effect. Fan et al. studied the damping effect of different thicknesses of shock absorbing layer and found that when the ratio of buffer layer thickness to inner diameters of secondary lining is 1/40 similar to 1/20, the shock-absorbing performance is remarkable [32]. Yang et al. [33] compared the model test with or without the damping layer, concluding that the damping layer could absorb seismic waves and thus reduce the interaction between surrounding soils and tunnel structure. 

Foam concrete or foam Aluminium are used in impact and shocking engineering so as to protect the structure [34,35,36,37]. They can be considered as a porous material that is able to absorb impaction and shocking by deformation. However, the tunnel construction process cannot easily accept them in practice, as they are not user friendly at this stage, especially the viscosity and fluidity. Rubber concrete is also a material used in civil engineering, which shows good damping effect [38,39,40]. However, rubber concrete is not a porous material and is not able to sustain large deformation such as the porous material. According to Lu et al. [41], the UCS for the grouting material to form the lining layer should be over 0.5 MPa for 3 days and 2.5 MPa for 28 days. However, there is no requirement for the flexural stiffness according to authors’ knowledge. 

Based on the concept of porous material and rubber concrete, a porous sand and rubber is introduced to concrete to form a possible grouting concrete. Consequently, tunnel construction can use the pumping method to fulfil the material. Preliminary evaluations of the grouting concrete were assessed to understand the basic effect of each ingredient (i.e., porous sand, rubber, cement) in the grouting concrete. 

## 2. Materials

The raw materials used in this study include porous sand, solid sand (i.e., quartz), rubber, and cement. The properties of the material were either given by the supplier or determined in the laboratory. 

### 2.1. Porous Sand

**Figure 1:**
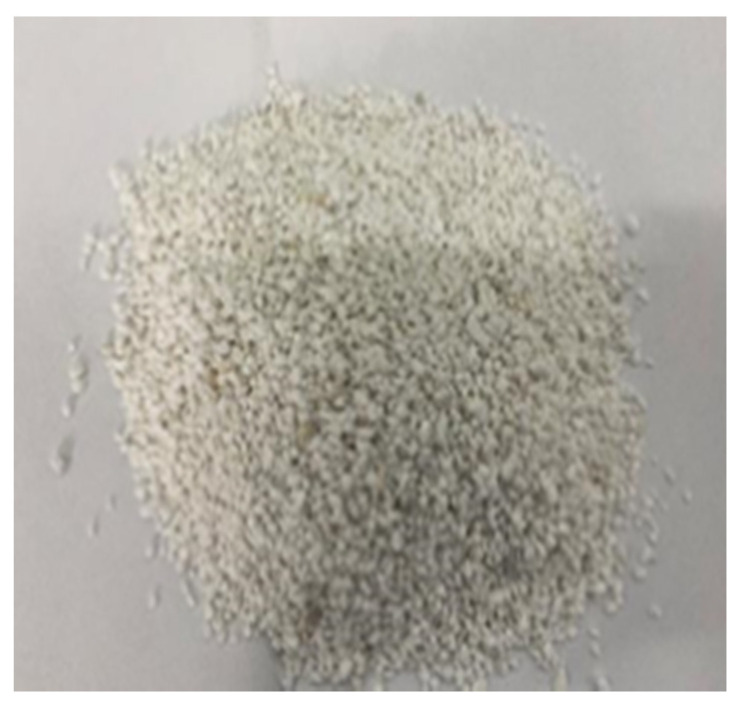
Profile of the white porous sand.

The porous sand (seen in Figure 1) used in this study was provided by Guangzhou Haizhenda Building Materials Co., Ltd., located in Guangzhou, Guangdong Province. The particle size of the porous sand ranges from 2 mm to 4 mm, with a sand grain density of 0.18 g/cm^3^ and a maximum water absorption rate of 43%. As a porous material, the porous sand contains a large number of pores. Currently, porous sand is mainly used as an insulation material. Due to its high number of internal pores, it has relatively low strength. Therefore, under external loads, its internal structure may be damaged, while also possessing energy-absorbing characteristics. Based on these properties, this study used porous sand as aggregate to prepare a new type of grout material.

### 2.2. Solid Sand (Quartz)

**Figure 2:**
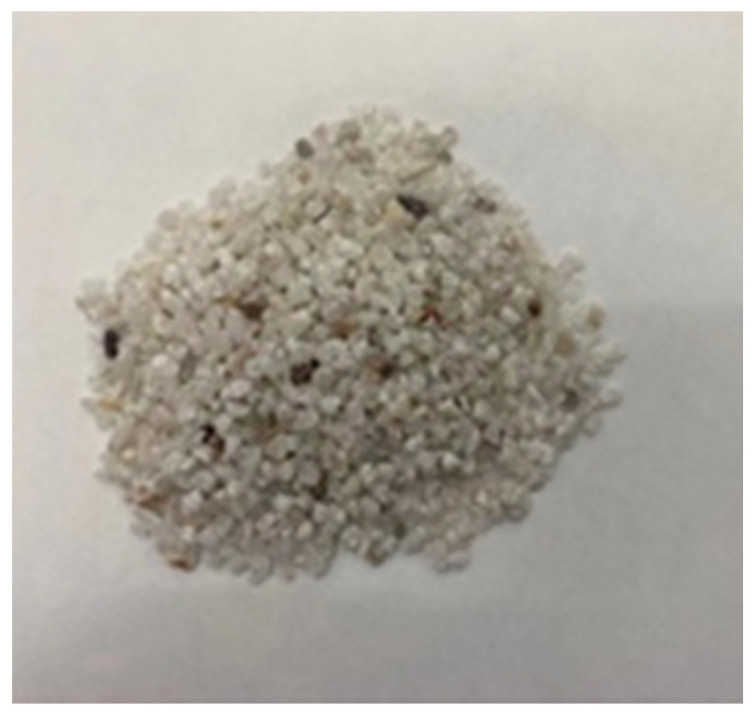
Profile of the solid sand (quartz).

Solid sand is currently the most widely used aggregate in concrete due to its high strength and stable performance. However, in some special constructions, it is often challenging for solid sand to meet the required standards based on different architectural requirements. The solid sand used is ordinary solid sand produced by Guangdong Yue Minerals Corporation. It originates from Heyuan, Guangdong, with a particle size ranging from 2 mm to 4 mm (refer to Figure 2). The particle density of the solid sand is 2.65 g/cm^3^. The composition of the solid sand can be found in Table 2.

### 2.3. Rubber

**Figure 3:**
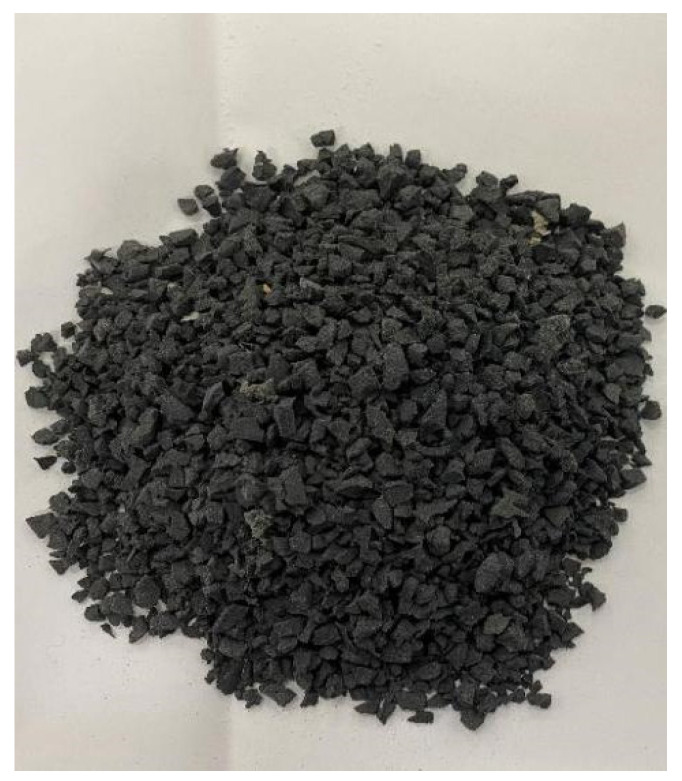
Profile of the rubber particle.

The rubber particles used in this study were produced by Rongchuang Building Materials Co., LTD., Lingshou County, Shijiazhuang, Hebei Province, China, with a density of 1.06 g/cm^3^ (Figure 3) and a size of 2 mm to 4 mm (provided by the supplier). This rubber is made from scrap tires, and the wire is removed from this rubber. Rubber is a high damping material. Because of its special viscoelasticity, it is often used as a shock-absorbing and energy-absorbing material in construction projects.

### 2.4. Cement

**Figure 4:**
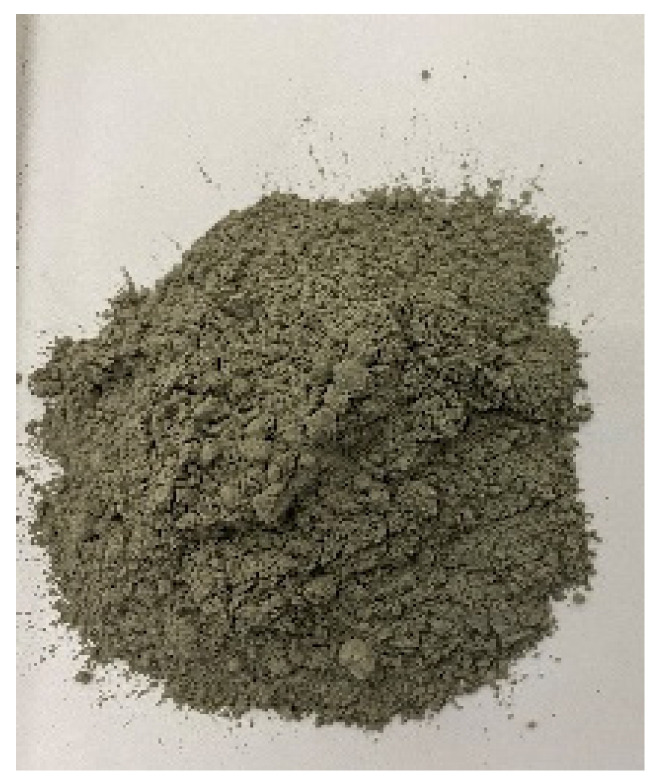
Profile of the cement.

Cement is an important building material, mainly used in the manufacture of concrete, mortar, and other building materials. The cement used in this study is produced by Guangzhou Yonggu Building Materials Co., Ltd. in Guangzhou, Guangdong Province. Cement can be mixed with lime or sand to make mortar or mortar, which is used to bond or fill building materials such as stone, brick, and tile. The cement is ordinary Portland cement (Figure 4), with a 3-day compressive strength of 24 MPa and a 28-day compressive strength of 47 MPa. Table 3 lists this combination.

## 3. Test Program

The volume in terms of sand and rubber is the same for Group A and B. Table 4 illustrates the key tests performed in this study. Table 5 lists the test conditions and mixing ratios for different groups. Each test had two identical specimens for repeatability. Group A evaluated the effects of different rubber contents, Group B compared the effect of porous sand with solid sand, and Group C evaluated the different water to cement ratio on the mechanical behaviour. The rubber content in this study was the volume of rubber to the total volume of rubber and sand. The calculation of volume needs to use the particle density which was mentioned in the previous section. 

### 3.1. UCS and Compaction Test

**Figure 5:**
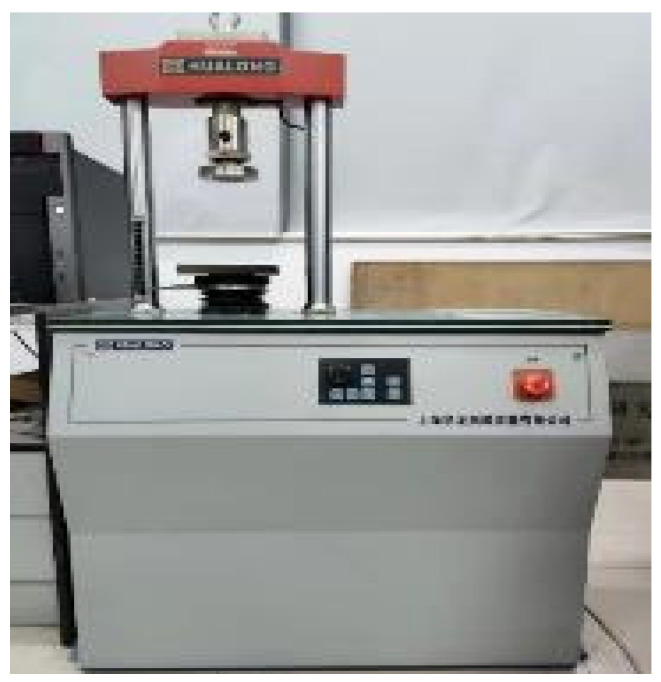
Profile of UCS test.

**Figure 6:**
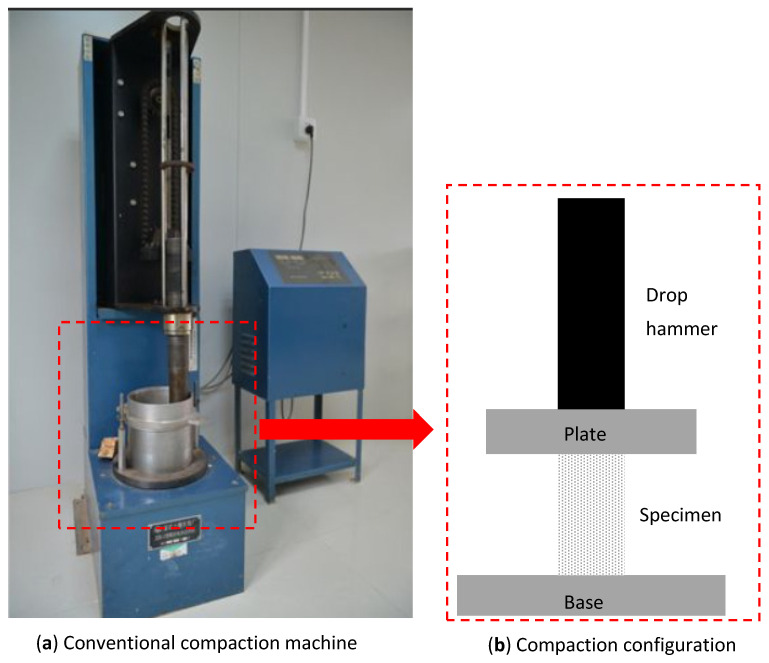
Profile of compaction test.

According to Table 5, cement, sand, and rubber were mixed evenly in a mixer to ensure a relatively good distribution of the materials [41]. Then, the target amount of water was added to the mixture and mixed for 15 min. The room temperature and test condition were controlled at 20 ± 1 °C. The mixture was then poured into the mould and lightly compacted to form a cylinder specimen and cured for unconfined compression strength (UCS) test and compaction test according to ASTM D2166 [42] and D698 [43], respectively. The cylinder specimen size was 50 mm in diameter and 100 mm in height. The curing condition was 20 ± 1 °C and 100% in relative humidity. The loading rate was set at 0.6 mm/min for UCS (Figure 5). In the compaction test, a conventional compaction testing machine is used, specifically the 50 kN press machine produced by Shanghai MeiYu Instrument Technology Co., Ltd. (Shanghai, China) (refer to Figure 6).

**Table 5:** Test conditions and mixing ratio in this study.

Group	Test No.	Water (g)	Cement (g)	Rubber (g)	Sand (g)	Sand Type	Test Type ^(a,b)^	Rubber Content ^(c)^ (%)	Water to Cement Ratio
A	1	1	1.60	0.00	0.26	Porous	UCS, F, C	0	0.6
	2	1	1.60	0.31	0.21	Porous	UCS, F, C	20	0.6
	3	1	1.60	0.62	0.16	Porous	UCS, F, C	40	0.6
	4	1	1.60	0.93	0.11	Porous	UCS, F, C	60	0.6
	5	1	1.60	1.24	0.05	Porous	UCS, F, C	80	0.6
B	1	1	1.60	0.00	3.90	Solid	UCS	0	0.6
	2	1	1.60	0.31	3.10	Solid	UCS	20	0.6
	3	1	1.60	0.62	2.33	Solid	UCS	40	0.6
	4	1	1.60	0.93	1.55	Solid	UCS	60	0.6
	5	1	1.60	1.24	0.78	Solid	UCS	80	0.6
C	1	1	1.67	0.93	0.11	Porous	UCS, F, C	60	0.6
	2	1	1.43	0.93	0.11	Porous	UCS, F, C	60	0.7
	3	1	1.25	0.93	0.11	Porous	UCS, F, C	60	0.8
	4	1	1.11	0.93	0.11	Porous	UCS, F, C	60	0.9
	5	1	1.00	0.93	0.11	Porous	UCS, F, C	60	1

^a^: UCS = Unconfined Compressive Strength, F = Flexural Strength, C = Compaction; each has another parallel test. ^b^: Curing time for UCS is 7 days and 28 days and for F and C is 7 days. ^c^: Rubber content = volume of rubber to the total volume of rubber and sand.

**Figure 7:**
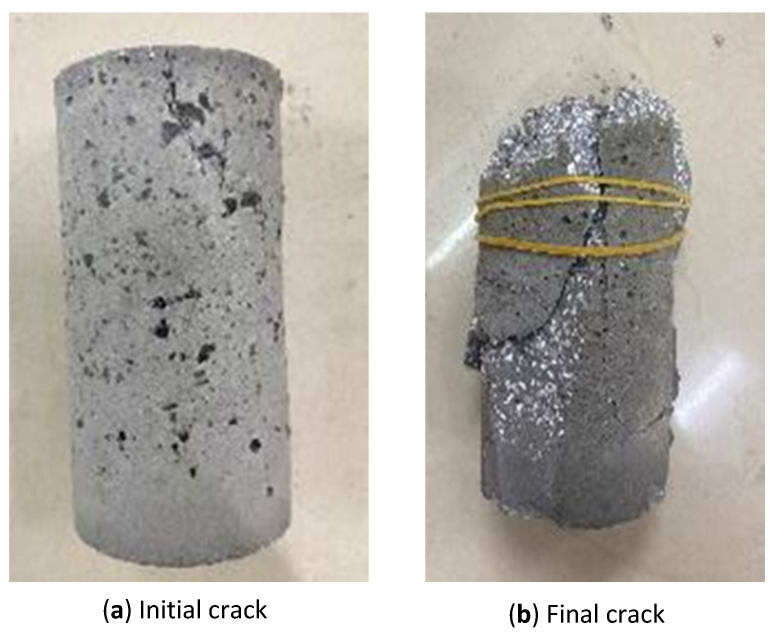
Definition of initial and final crack.

The drop hammer tester was produced by Yangzhou Yuanfeng Testing Equipment Co., Ltd. in Yangzhou City. It was a drop-weight impact testing machine, as shown in Figure 6. The weight of the hammer was 4.5 kg, and the height of the drop was 355 mm, so the accumulative energy could be calculated. After each drop, observation was made on the specimen. When the first crack appeared on the surface of the specimen (Figure 7), the corresponding number of drop (i.e., the accumulative energy) was defined to form the initial crack. The specimen was then continuously impacted until it was damaged, which was defined as its expansion in the radial direction by 10% (i.e., 5 mm in the radial direction) and measured by a calliper (Figure 7). 

### 3.2. Flexural Stiffness Test

**Figure 8:**
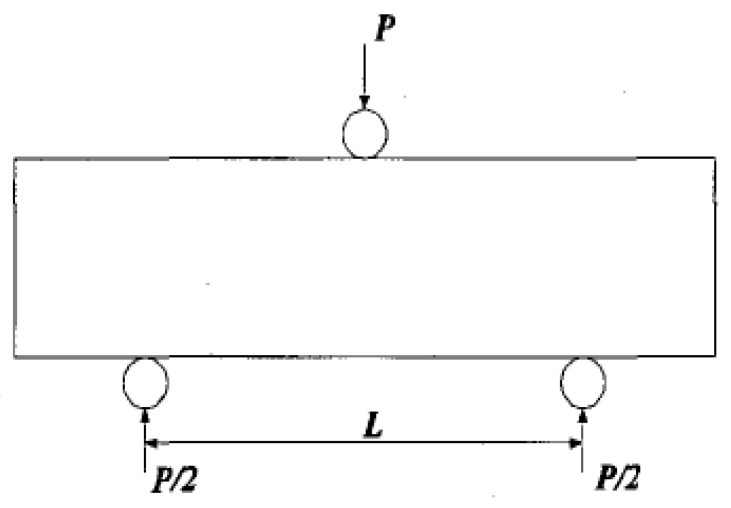
Three points method for flexural strength test.

Similar preparation method was used for the flexural stiffness test (i.e., three points method shown in Figure 8) according to ASTM C78 [44]. The mixture was poured into the mould (40 mm × 40 mm × 160 mm) and lightly compacted. The curing condition was also 20 ± 1 °C and 100% in relative humidity. The loading rate was set as 0.6 mm/min as well. 

## 4. Results and Discussions

The main ingredients of the proposed grouting material are porous sand, rubber, and cement, and each of them is expected to have particular role to design the material. The porous sand is to provide pore volume, the rubber is to provide elasticity, and the cement is to provide cementation and bonding strength. 

### 4.1. Effect of Porous Sand

The idea of the grouting concrete proposed in this study was learnt from foam concrete or foam aluminium [34,35,36,37,45]. They are considered as porous materials and usually applied in the impact engineering to protect the structure [46,47,48,49]. The mechanism of a tunnel to resist dynamic loading and large deformation (i.e., creep) is similar to the impact engineering, so porous material has great engineering potential to apply in tunnel construction. However, the pumping technique for both materials is difficult to apply in practice [50]. Thus, to be easily acceptable for the conventional tunnel construction (e.g., shield tunnel), the pumping technique is more preferred to apply. 

**Figure 9:**
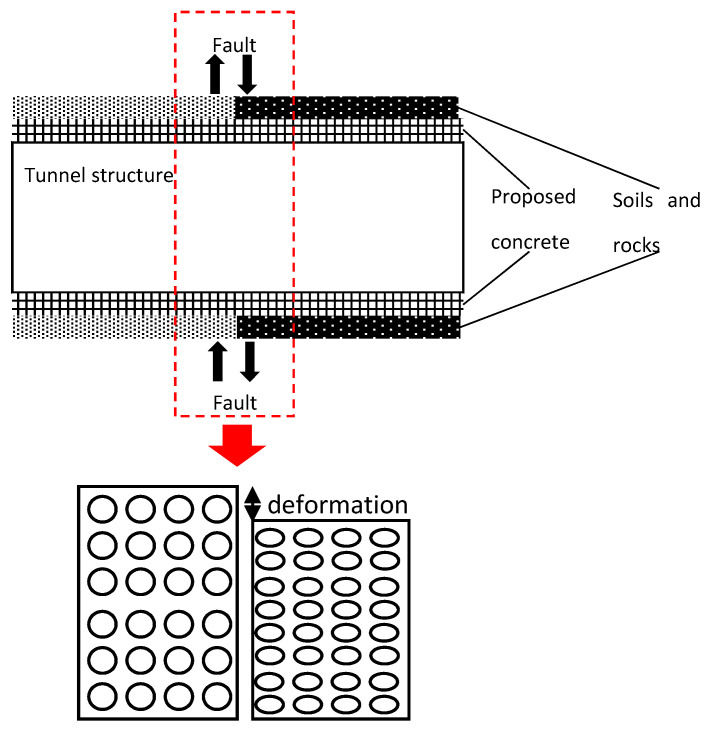
Configuration of proposed concrete deformed under slow movement.

The porous sand added is to provide pore volume (Figure 9), so that the material with porous sand can be compressed vertically. This characteristic will allow the tunnel to be constructed at the active rock or across the fault which usually undergo large but slow movement. In this case, the strength of the material is deemed relatively low (e.g., 1~2 MPa). When large but slow movement is triggered, the material is expected to be crushed first and then to fill the pore volume, which is similar to the foam concrete and foam aluminium.

**Figure 10:**
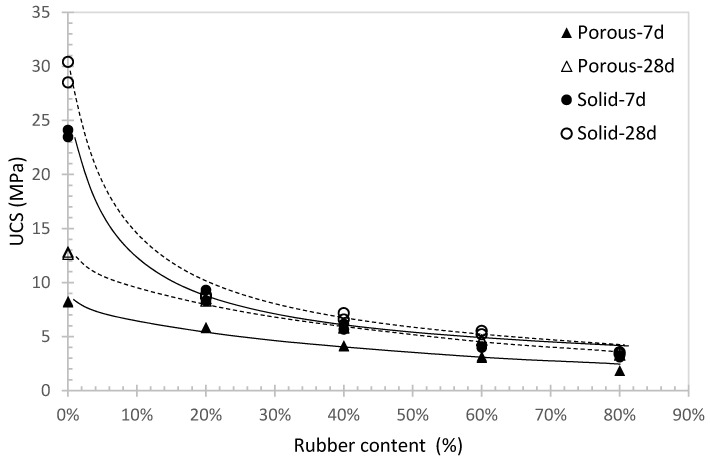
UCS comparisons between porous and solid sand at water to cement ratio of 0.6.

Figure 10 demonstrates the UCS against rubber content for solid and porous sand at the same mixing condition. It can be observed that UCS decreases as the rubber content increases, which is consistent with many other studies in the literature. Additionally, the UCS for solid sand is generally higher than that for the porous sand. In fact, the mechanism of the two aggregates is different. When solid sand is added, the material is going to be compressed without pore volume to be filled. This will result in high compressive strength of the concrete. However, to be a satisfied grouting material for tunnelling, the protective layer needs to have a relatively low UCS and able to sustain large deformation to protect the tunnel structure as explained in the early section. Thus, the solid sand is needed to be replaced by the porous sand which contains pore volume and venerable under compression. Consequently, it will lead to a relatively low UCS as shown in Figure 10, which is more preferred as the protective layer for tunnel construction. 

**Figure 11:**
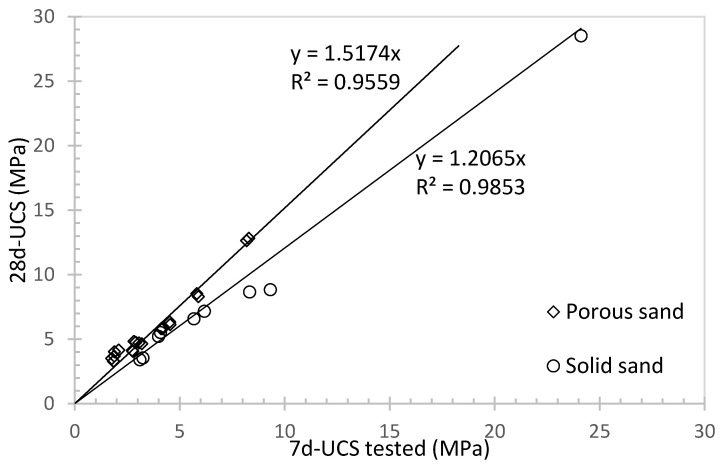
UCS comparisons between 7 and 28 days curing at water to cement ratio of 0.6 and different rubber content.

By comparing the data of porous sand at a water to cement ratio of 0.6 (i.e., groups A and C) with the UCS data of solid sand (i.e., group B) and integrating them, it was found that both materials exhibited linear relationship for predicting the 28-day UCS value. At the same 7-day UCS value, the 28-day UCS value of porous sand is usually higher than that of solid sand (Figure 11), but different mixing ratios are required to achieve the same 7-day UCS value. These results can provide some references and guidance for the application of porous materials in concrete structures.

### 4.2. Effect of Rubber

**Figure 12:**
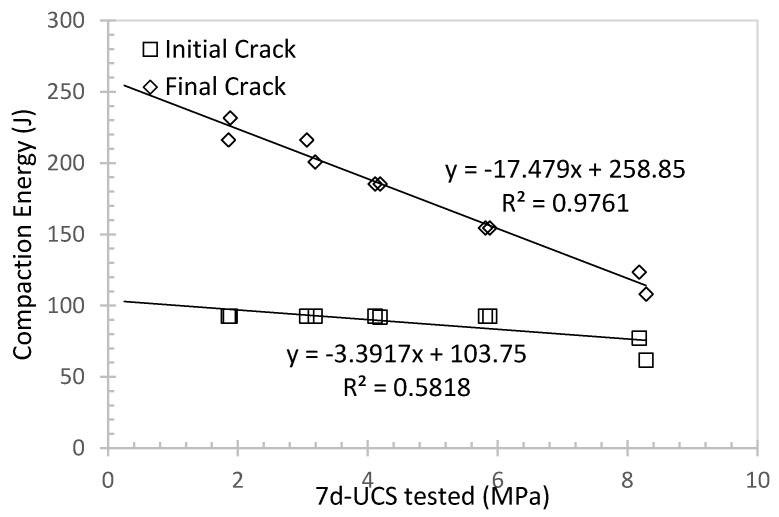
Compaction energy against 7d-UCS for porous sand at water to cement ratio of 0.6 and different rubber content.

As mentioned in the earlier section, increasing the rubber content will lower the UCS, and the advantage is to provide elasticity so as to reduce the wave transmission to the tunnel structure. Another advantage is to reduce the mechanical vibration from the subway or vehicles to the surrounding structures or buildings. Figure 12 summarises the relationship between the compaction energy and 7d-UCS for porous sand at water to cement ratio of 0.6 and different rubber contents. As the UCS increased due to less rubber content presenting in the specimen, less energy was needed to break the specimen. This behaviour is consistent with many other studies [51]. As rubber can be considered a good damping material to absorb energy [52], less rubber content available in the specimen indicates less impaction resistance to absorb the energy. It can be also observed from Figure 12 that the energy needed to initiate the initial crack and final crack can be both predicted with the 7d-UCS in the linear form. A rough estimation can be given based on 7d-UCS.

### 4.3. Effect of Cement

**Figure 13:**
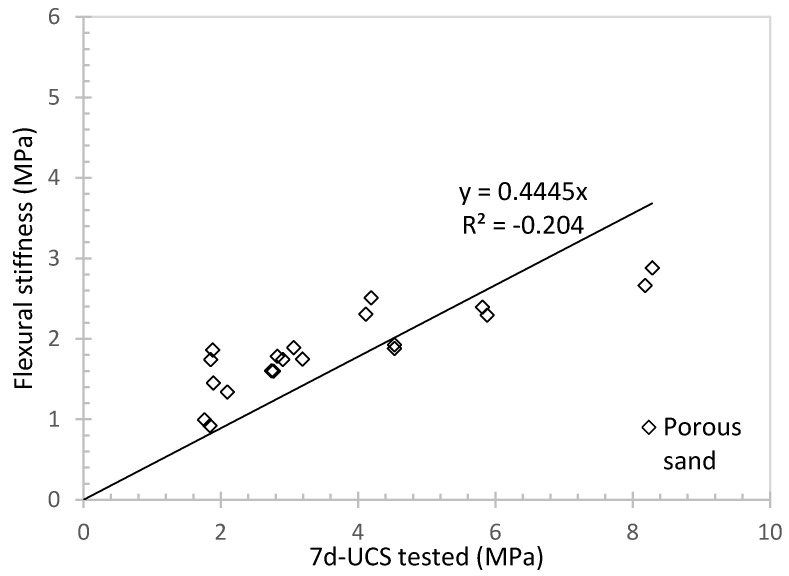
Flexural stiffness against 7d-UCS for porous sand at different water to cement ratio and rubber content.

**Figure 14:**
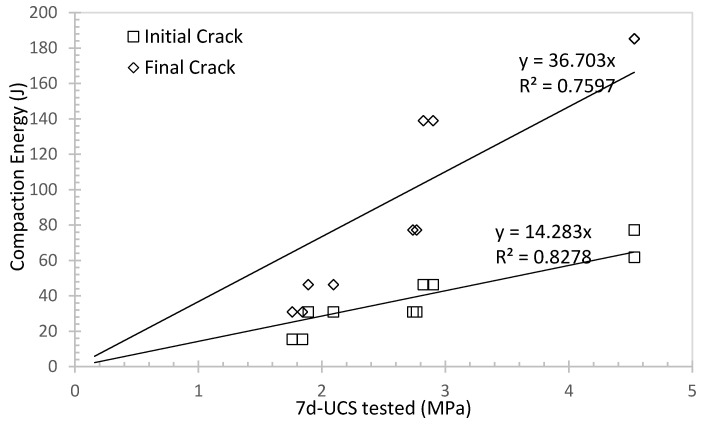
Compaction energy against 7d-UCS for porous sand at 60% rubber content and different water to cement ratio.

The cement in this material is used to increase the bonding strength and provide cementation. Evaluations were conducted using flexural stiffness and compaction tests. Flexural stiffness is an important parameter used to define a material’s mechanical behaviour against bending moment [53]. Figure 13 demonstrates the linear relationship between flexural stiffness and 7d-UCS obtained by integrating the data from Groups A and C. Similarly, Figure 14 summarizes the relationship between the compaction energy and 7d-UCS for porous sand at 60% rubber content and varying water-to-cement ratios. As the water-to-cement ratio increases and cementation effect becomes stronger, larger UCS values are obtained which requires more energy to break the specimen. Additionally, both initial and final cracks show a linear relationship with 7d-UCS. As a result, this relationship will help in making quick estimations. Overall, both flexural stiffness and compaction tests indicate that cement only provides cementation and bonding strength to the material, and more energy is required to break the bonding strength in case of strong cementation.

## 5. Prediction of 7d-UCS

**Figure 15:**
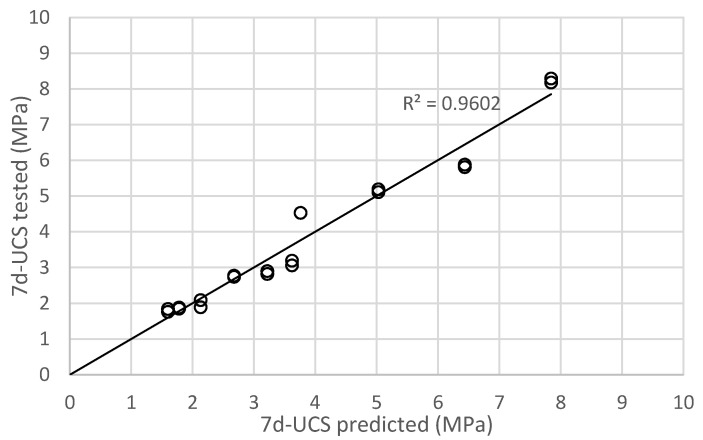
Comparisons between 7d-UCS predicted and tested result.

As demonstrated in the previous section, the porous sand, rubber, and cement with different mechanical behaviour (e.g., UCS, flexural stiffness, compaction) are directly related to 7d-UCS. Thus, 7d-UCS is important to know in order to estimate other mechanical behaviour such as the flexural stiffness. Based on this feature, the software, IBM SPSS Statistics, was used in this study to carry out multiple linear regression on the test data and to predict 7d-UCS. The linear relationship was assumed in the prediction process. In this study, water to cement ratio (w/c), rubber mass ratio (R), and porous sand mass ratio (P) were defined as independent variables, and UCS was defined as dependent variable. After the data in Group A and C were input to the SPSS, the results of multiple linear parameters were obtained. Table 6 summarised the key information from SPSS simulation. It can be observed that R^2^ was 0.966 in the regression simulation results. Statistically, R^2^ larger than 0.3 indicates that independent variables can express the changes of dependent variables through the formula, so this regression model is acceptable. The significance of the coefficient for w/c, R, and P was 0.001 and lower than 0.05. This means that all three independent variables can significantly affect UCS. Meanwhile, the coefficient for w/c was −5.391, for R was 2.415, and for P was 43.122. The significance for constant term is 0.989, indicating the constant term has negligible effect on the dependant variable (i.e., UCS). So, the constant term is ignored in this study, and the final form to predict 7d-UCS is shown in Equation (1). Figure 15 compares the predicted and actual UCS, and the overall performance is reasonably good.

(1) UCS=−5.391×w/c+2.415×R+43.122×P

where UCS is 7d-UCS (MPa); w/c is water to cement ratio; R is rubber mass ratio in Table 5; and P is porous sand mass ratio in Table 6.

However, it needs to be mentioned that Equation (1) is currently only valid for this particular cement that comprises the specific porous sand. This assumes that the effect of porous sand is the same. More experiments need to be conducted to verify. 

## 6. Conclusions

The main difference of the proposed concrete is the introduction of porous sand to the concrete that allows the concrete to sustain large but slow deformation and reduce the mechanical vibration to the surrounding structure. The results show that the UCS is dropped significantly when replacing the solid sand with porous sand as the rubber content is increased. Similarly, when the rubber content increases, the UCS of the material decreases, which is consistent with many studies. The increase in the rubber content helps the material to gain a resistance against compaction. Additionally, the relationship of energy required has a linear relationship with 7d-UCS. Thus, 7d-UCS is an important parameter to know and estimate. Consequently, the mathematical equation to predict 7d-UCS is proposed, which can be used in practice to quickly predict the compaction energy and UCS. Overall, the test results are consistent with the fundamental mechanism of the tunnel construction and provide positive feedback of the material. However, the proposed material needs further in-depth study in terms of workability, durability, etc., before application being undertaken. 

## Figures and Tables

**Table 1:** Examples of major earthquake damage in tunnels and underground engineering.

Case of Earthquake Damage	Time	Locations	Result
Tokyo earthquake (M7.9)	1923	Japan	Twenty-five tunnels in the nearby city suffered varying degrees of damage, with fourteen mainly suffering damage to their structures. Some of the tunnels have experienced damage due to arch collapse and extensive slope collapse at their entrance.
Izu earthquake (M7.0)	1930	Japan	As a result of the earthquake, the horizontal dislocation of the drainage tunnel in the Shidona railway tunnel under construction exceeded 2 m, and the vertical dislocation reached 0.6 m. Numerous cracks appeared on the side wall of the tunnel.
Izu-o earthquake (M7.0)	1978	Japan	In the Inatvri tunnel area, a large crack has appeared, causing damage to the central area of the tunnel. The invert and initial lining of the tunnel suffered severe cracking, and the concrete within the tunnel has spalled in numerous locations.
Earthquake in southern Hyogo Prefecture (M7.2)	1995	Japan	Approximately 10% of the mountain tunnel sustained extremely serious damage that requires varying degrees of strengthening and repair after the earthquake. The mountain tunnel located in the harsh geological section suffered lining collapse.
Jiji earthquake	1999	Taiwan (China)	Several tunnels suffered severe damage, with 50 tunnels on the fault and east side being affected. The most significant damage was to the lining structure of the Sanyi No. 1 railway tunnel, which traverses the west side, leading to the disruption of rail traffic for 18 days.
Wenchuan earthquake (M8.0)	2008	China	The epicentre of the earthquake was located 12–15 km away, and 80.9% of the 52 highway tunnels in Sichuan sustained varying degrees of damage. The roads leading to the disaster area were almost completely disrupted.

**Table 2:** Composition for solid sand (quartz) given by the supplier.

Composition	SiO_2_	TiO_2_	Al_2_O_3_	CaO	Na_2_O	Fe_2_O_3_	K_2_O	MgO
Content (%)	68.91	0.31	9.50	5.33	4.00	2.26	5.08	2.86

**Table 3:** Composition for cement given by the supplier.

Ingredient	CaO	SiO_2_	Al_2_O_3_	Fe_2_O_3_	MgO	SO_3_	Other	Ignition Lost
Content (%)	58.89	22.14	6.59	2.69	2.53	2.47	0.78	3.06

**Table 4:** Tests performed in this study.

UCS	ASTM D2166 [42]
Compaction	ASTM D698 [43]
Flexural stiffness	ASTM C78 [44]

**Table 6:** Results of SPSS computation.

Independent Variable	Coefficient	Significance	R^2^
Water to cement ratio (w/c)	−5.391	≤0.001	0.966
Rubber mass (R)	2.415	≤0.001	
Porous sand mass (P)	43.122	≤0.001	
Constant	0.006	0.989	

## Data Availability

All data that support the findings of this study are included within the article.
